# The real seroprevalence of SARS-CoV-2 in France and its consequences for virus dynamics

**DOI:** 10.1038/s41598-021-92131-0

**Published:** 2021-06-15

**Authors:** Chloé Dimeglio, Jean-Michel Loubes, Marcel Miedougé, Fabrice Herin, Jean-Marc Soulat, Jacques Izopet

**Affiliations:** 1 2 INSERM UMR1291 – CNRS UMR5051, Toulouse Institute for Infectious and Inflammatory Diseases (INFINITy), 31300 Toulouse, France; 2 Virology Laboratory, CHU Toulouse, Hôpital Purpan, 31300 Toulouse, France; 3 Institut de Mathématiques de Toulouse, Université de Toulouse, 31400 Toulouse, France; 4 Occupational Diseases Department, Toulouse-Purpan University Hospital, 31000 Toulouse, France

**Keywords:** SARS-CoV-2, Epidemiology, Statistical methods

## Abstract

The SARS-CoV-2 virus has spread world-wide since December 2019, killing more than 2.9 million of people. We have adapted a statistical model from the SIR epidemiological models to predict the spread of SARS-CoV-2 in France. Our model is based on several parameters and assumed a 4.2% seroprevalence in Occitania after the first lockdown. The recent use of serological tests to measure the effective seroprevalence of SARS-CoV-2 in the population of Occitania has led to a seroprevalence around 2.4%. This implies to review the parameters of our model to conclude at a lower than expected virus transmission rate, which may be due to infectivity varying with the patient’s symptoms or to a constraint due to an uneven population geographical distribution.

## Introduction

The severe acute respiratory syndrome coronavirus 2 (SARS-CoV-2) that emerged in Wuhan, China in December 2019 has spread largely by sustained human-to-human transmission^1^. The WHO declared the resulting disease a pandemic^2^. The virus, which causes severe respiratory illness in susceptible individuals, has spread so rapidly that it has led most affected countries to lockdown their populations in order to break the dynamics of the virus’s evolution. Many models have used the proportion of SARS-CoV-2 infected people tested by RT-PCR tests to predict the course of the epidemic^3–5^. Our statistical model for predicting the spread of SARS-CoV-2 in France is based on a diffusion and transmission coefficient that varies with an individual’s age, the likelihood of contagion, and two administration parameters (lockdown and quarantine)^4^. It indicated that the seroprevalence in France at the end of the first lockdown (from March 17, 2020 to May 11, 2020) would be no greater than 17.5%^4^. Recent results of serological tests measuring the effective seroprevalence of SARS-CoV-2 in the general population have led us to assess the consequences of a much lower seroprevalence on the real virus dynamics in the population. The aim of this work is to determine the reason why there is a difference between the seroprevalence results obtained by the model and those obtained by the serological tests, and to modify the parameters of the model accordingly.

## Results

We define $I_{{d,R}} ~{\text{as}}~$ the real seroprevalence on day $d$ at the end of lockdown and $I_{{d,E~}}$ as the seroprevalence estimated by the model on the same day. More generally, for a given variable $V$, $V_{R}$ is the observed variable and $V_{E}$ is the variable estimated by the model.

We assumed that there were 3 untested SARS-CoV-2-contagious people in France when the first 3 cases were detected on January 24 (50% of asymptomatic cases). We assume that the number of tests carried out $(\alpha$) has remain fixed, since the virology laboratories have never been saturated, and that the number of days a person is contagious $N_{T}$ is equal to 20^6^.

The quarantine constraint $q$ is considered to be negligible.

### Seroprevalence

Lockdown began on March 17 in France and ended on May 11. The predicting model indicated that the seroprevalence in Occitania before March 17 was 0%, and that it was 4.2% after lockdown. From June 10, 2020 to July 10, 2020, 1 to 2 months after the end of lockdown in France, everyone who worked at Toulouse University Hospital (n = 16,571) was invited for screening total serum antibodies by ELISA test. Among the 8758 people who took part in the study, the ELISA test indicated that 276 had SARS-CoV-2 antibodies, corresponding to an overall seroprevalence of 3.2% (95% confidence interval [CI] 2.8–3.5%)^7^. Based on the demography in the Occitania region, the median age was 44 years (IQR 30–57) for 58.2% of women and 42.8% of men. Corrections were performed using the R software/environment^8^ version R-3.5.1. (Copyright 2007 Free Software Foundation, Inc; http://www.gnu.org/licenses/gpl.html). We have used the “sampling” package obtained from the Comprehensive R Archive Network (CRAN) mirror sites^9^ to correct the estimated SARS-CoV-2 seroprevalence based on our dataset^10^. By correcting our seroprevalence data to align them with these regional demographic data and the geographical distribution of postcodes, we obtained a SARS-CoV-2 seroprevalence of 2.4%.

Consequently, it is quite possible that the national seroprevalence of SARS-CoV-2 is also lower than expected.

### Consequences for SARS-CoV-2 dynamics

Since the real seroprevalence is lower than expected on day $d$ at the end of lockdown, we have

1 $$I_{{d,~R}} < ~I_{{d,E}} .$$

According to Eq. (4), the number of immunized people on day $~d$ corresponds to the number of people immunized on day $d - 1$ to which we add the people who were on their last day of infection on day $d - 1$, whether they were tested or not.

We have $I_{{d,R}} = \left( {I_{{d - 1}} + P_{{d - 1}}^{{20}} + ~Q_{{d - 1}}^{{20}} } \right)_{R}$, $I_{{d - 1,R}} = \left( {I_{{d - 2}} + P_{{d - 2}}^{{20}} + ~Q_{{d - 2}}^{{20}} } \right)_{R}$ and finally, $I_{{d,R}} = \left( {I_{1} + \mathop \sum \nolimits_{{n = 1}}^{{d - 1}} P_{n}^{{20}} + \mathop \sum \nolimits_{{n = 1}}^{{d - 1}} Q_{n}^{{20}} } \right)_{R}$ with $I_{1} = 0$ (nobody immunized on day one).

Equation (1) becomes $$\left( {\mathop \sum \nolimits_{{n = 1}}^{{d - 1}} P_{n}^{{20}} + \mathop \sum \nolimits_{{n = 1}}^{{d - 1}} Q_{n}^{{20}} } \right)_{R} < ~\left( {\mathop \sum \nolimits_{{n = 1}}^{{d - 1}} P_{n}^{{20}} + \mathop \sum \nolimits_{{n = 1}}^{{d - 1}} Q_{n}^{{20}} } \right)_{E} .$$

Since $$\forall ~n < 20,~P_{n}^{{20}} = 0~and~Q_{n}^{{20}} = 0$$, (1) becomes

$$\left( {\mathop \sum \limits_{{n = 20}}^{{d - 1}} P_{n}^{{20}} + \mathop \sum \limits_{{n = 20}}^{{d - 1}} Q_{n}^{{20}} } \right)_{R} < ~\left( {\mathop \sum \limits_{{n = 20}}^{{d - 1}} P_{n}^{{20}} + \mathop \sum \limits_{{n = 20}}^{{d - 1}} Q_{n}^{{20}} } \right)_{E} .$$

According to Eq. (2), the number of undetected contagious carriers on day $n$ is the number of undetected carriers who were infected and undetected on day $n - 1$ and who were not tested.

We have:

$$\begin{array}{*{20}c} {P_{n}^{{20}} = ~P_{{n - 1}}^{{19}} ~\left( {1 - \alpha } \right),} \\ {P_{{n - 1}}^{{19}} = ~P_{{n - 2}}^{{18}} ~\left( {1 - \alpha } \right),} \\ \vdots \\ \end{array}$$

Finally, $P_{n}^{{20}} = ~P_{{n - 19}}^{1} ~\left( {1 - \alpha } \right)^{{19}}$ with $P_{{n - 19}}^{1}$ defined by Eq. (3).

Similarly, $Q_{n}^{{20}}$ is defined by Eq. (5) as the number of detected contagious carriers on day $n - 1$ to which we add the number of undetected contagious carriers on day $n - 1$ who were tested.

We have:

$$\begin{array}{*{20}c} {Q_{n}^{{20}} = ~Q_{{n - 1}}^{{19}} + P_{{n - 1}}^{{19}} ~ \cdot \alpha \;{\text{with}}\;Q_{{n - 1}}^{{19}} = ~Q_{{n - 2}}^{{18}} + P_{{n - 2}}^{{18}} ~ \cdot \alpha ,} \\ {Q_{n}^{{20}} = ~Q_{{n - 2}}^{{18}} + P_{{n - 2}}^{{18}} ~ \cdot \alpha + P_{{n - 2}}^{{18}} ~ \cdot \alpha \cdot ~\left( {1 - \alpha } \right)} \\ \vdots \\ \end{array}$$

Finally, $Q_{n}^{{20}} = ~Q_{{n - 19}}^{1} + P_{{n - 19}}^{1} \cdot \mathop \sum \nolimits_{{m = 0}}^{{18}} \alpha \cdot ~\left( {1 - \alpha } \right)^{m}$ with $Q_{{n - 19}}^{1} = 0~$(no quarantine on day one) and $P_{{n - 19}}^{1}$ defined by Eq. (3).

Equation (1) becomes:

$$\left( {\mathop \sum \limits_{{n = 20}}^{{d - 1}} \frac{{S_{n} }}{N} \cdot \left[ {\mathop \sum \limits_{i} P_{{n - 20}}^{i} \cdot R_{0}^{i} \cdot c~} \right] \cdot \left[ {\mathop \sum \limits_{{m = 0}}^{{18}} \alpha \cdot ~\left( {1 - \alpha } \right)^{m} + \left( {1 - \alpha } \right)^{{19}} } \right]} \right)~_{R} < \left( {\mathop \sum \limits_{{n = 20}}^{{d - 1}} \frac{{S_{n} }}{N} \cdot \left[ {\mathop \sum \limits_{i} P_{{n - 20}}^{i} \cdot R_{0}^{i} \cdot c~} \right] \cdot \left[ {\mathop \sum \limits_{{m = 0}}^{{18}} \alpha \cdot ~\left( {1 - \alpha } \right)^{m} + \left( {1 - \alpha } \right)^{{19}} } \right]} \right)~_{E} ,$$

which equals:

$$\left( {\mathop \sum \limits_{{n = 20}}^{{d - 1}} \frac{{S_{n} }}{N} \cdot \left[ {\mathop \sum \limits_{i} P_{{n - 20}}^{i} \cdot R_{0}^{i} \cdot c~} \right]} \right)~_{R} < \left( {\mathop \sum \limits_{{n = 20}}^{{d - 1}} \frac{{S_{n} }}{N} \cdot \left[ {\mathop \sum \limits_{i} P_{{n - 20}}^{i} \cdot R_{0}^{i} \cdot c~} \right]} \right)~_{E} .$$

The three possible explanations for why the real seroprevalence is lower than expected are:

1. $c_{R} < c_{E}$ i.e. the real lockdown constraint $c_{R}$ was more stringent than expected $c_{E} .$
2. $P_{{n,R}} < ~P_{{n,E}}$ i.e. the fraction of the population initially infected but not detected $P_{{n,R}}$ was smaller than expected $P_{{n,E}}$.
3. $\left( {R_{0}^{i} } \right)_{R} < ~\left( {R_{0}^{i} } \right)_{E}$ i.e. the transmission rate was lower than expected.

## Discussion

We used a discretized SIR model to predict the dynamics of SARS-CoV-2 infections that takes into account various factors involved in the virus dynamics. We have assumed that the transmission of SARS-CoV-2 was identical regardless of the age of each individual or any individual characteristic. Although children could be less susceptible to SARS-CoV-2^11,12^, there is limited published evidence of age-related differences in infectivity^13–15^. Moreover, there may be errors in ascertaining the direction of transmission, leading to confusing differences in infectiousness with differences in susceptibility. Lockdown began on 17 March in France, and was gradually relaxed from May 11. Under these circumstances, the seroprevalence in Occitania at the end of lockdown was estimated to be 17.5% in France and 4.2% in Occitania^4^. But the serological tests carried out since the end of May indicate that the real seroprevalence is lower which suggests that the national seroprevalence is also lower. This is consistent with the findings of a study indicating that 4.4% (range 2.8% to 7.2%) of the French population (2.8 million people) had been infected^16^ and with the first results of the national study EpiCoV^17^ that found 4.5% of the French people had been affected by SARS-CoV-2 at the end of the first lockdown (May 11).

This could be because the actual lockdown constraint $c_{R}$ was more stringent than expected $c_{E}$. This seems unlikely since we have set the lockdown constraint at 80% and that it was neither strictly respected nor as restrictive as expected, given that approximately 29% of the French population continued to go to work during the first lockdown^18^. This mainly concerned healthcare workers who were required face-to-face for the management of the Covid-19 crisis and people who could not telework.

The second possibility is that the fraction of the population initially infected but not detected $P_{{n,R}}$ was smaller than expected, $P_{{n,E}}$, or that the asymptomatic people were less contagious than those who were symptomatic. The Center for Disease Control and Prevention (CDC) has reported that researchers in Singapore concluded that asymptomatic people were the source of 44% of diagnosed COVID-19 cases^19^. Another recent study reported that 104/128 (81 percent) of people on a cruise ship who tested positive for SARS-CoV-2 were asymptomatic^20^. And yet another study found that 42% of those who tested positive for SARS-CoV-2 were without symptoms^21^. On the contrary, we could also consider that the proportion of people infected but not detected was higher than that actually estimated due to the lack of sensitivity of the RT-PCR tests which can be negative for truly infected participants or early infected participants with waning immunity^22^. The proportion of asymptomatic cases in our model was close to 50% but it is possible that the actual proportion was lower even if the assumed proportion remains within the possible range. The expected proportion of asymptomatic people could be higher than that actually observed if these asymptomatic people were less contagious than expected. Our model assumes that the transmission rate can vary with the day of infection but is independent of the patient's symptoms. However, the proportion of asymptomatic infected people could also be lower than expected if these people were less contagious. This relationship was favored in a recent study looking at the symptoms of 455 cases who were in contact with a confirmed but asymptomatic SARS-CoV-2 patient^23^.

Our last explanation for a lower than expected seroprevalence assumes that the transmission rate was lower than expected: $\left( {R_{0}^{i} } \right)_{R} < ~\left( {R_{0}^{i} } \right)_{E}$. The WHO set this rate at an average of 2.2^24^, with occasional large variations. A recent review comparing 12 studies that estimated the $R_{0}$ for COVID- 19 found values of from 1.5 to 6.68^25^. The 2.2 average rate may be less important in reality or it may vary according to an individual’s symptoms, as previously discussed. It is also possible that the rate itself does not vary but that its constraints do. Our model assumes that the virus is uniformly distributed throughout the population, but the first episodes of virus infection occurred in clusters, which assumes concentrations of population in given places without necessarily any communication between the clusters. A recent analysis of the haplotypes of SARS-CoV-2 infections in Iceland concluded that the geographical distribution of clades was highly structured^26^.Thus the rate of virus transmission may be that set by the WHO within clusters but it could vary outside them or be much lower because of the uneven population density.

Serological testing has shown that the seroprevalence of SARS-CoV-2 is much lower than expected. In particular our prediction models assumed a 4.2% seroprevalence in Occitania and it turned out to be only 2.4%. This implies slower than expected virus transmission, perhaps due to variations in infectivity influenced by a patient’s symptoms or to geographical constraints on the population distribution. It will probably be necessary in the future to include a population density parameter in the SARS-CoV-2 diffusion models, or to work at equal densities^27^, to avoid bias.

## Methods

### Serological tests

Total plasma anti-SARS-CoV-2 antibodies (Ab) were detected using an enzyme linked immunosorbent assay (ELISA) kit (Beijing Wantai Biological Pharmacy Entreprise Co., Ltd, China) according to the manufacturer’s instructions. This assay is based on a recombinant antigen containing the SARS-CoV-2 spike protein receptor-binding domain (RBD). The sensitivity using samples collected 15 to 45 days post symptom-onset or after contact with a SARS-CoV-2 case, including asymptomatic patients, was 100% (CI 95% 88.2–100%) and the specificity using plasma collected before the outbreak of SARS-CoV-2 was 100% (CI 95% 82.1–100%)^28^. Human samples were taken after obtaining the informed consent for the SARS-CoV-2 surveillance. Biological material and clinical data were obtained only for standard viral surveillance (no specific sampling, no modification of the sampling protocol, no questions in addition to the standardized questionnaire). Data were analysed using an anonymized database. According to the Public Health French law (CSP Art L 1121-1.1), such a protocol does not require written informed consent. This study was entered in the Toulouse University Hospital register of retrospective studies: Rn IPH 2020-33 and is covered by MR-004 in accordance with the French data protection authority (Commission Nationale de l’Informatique et des Libertés—CNIL—number: 2206723 V0). All methods were carried out in accordance with relevant guidelines and regulations.

### Statistical model

The model is a discretized version of a Susceptible Infectious and Recovered (SIR)-type model^29^. We have added a diffusion/transmission coefficient $R_{0}$ in our model that varies with the likelihood of contagion, and two reduction coefficients $c$ and $q$ to describe the impact of public health measures on virus transmission in France.

We estimated the initial model settings using data published by Johns Hopkins University for France (Johns Hopkins database: https://github.com/CSSEGISandData/COVID-19 verified on 03/28/2020.) and data collected by the Toulouse Virology Laboratory. The total French population is N = 67,000,000 (source INSEE). SARS-CoV-2 screening began on January 24, 2020 and containment on March 17. We assumed that there were 50 untested SARS-CoV-2-contagious people in France on January 25, and 3 tested cases (source Johns Hopkins). Some values of c and the value of q have been estimated in previous works by correcting the values predicted by the model using real data collected by the Toulouse Virology Laboratory^4,27^. The model predicts how the SARS-CoV-2 virus would have evolved and projects the daily number of new positive cases. Its accuracy was evaluated at 86.1%^30^. By cumulative effect, we therefore obtain a projection of the seroprevalence of SARS-CoV-2 in France.

We considered the variables $\left( {S_{n} ,~P_{n} ,~Q_{n} ,~I_{n} } \right)$.

$S_{n}$ is the number of healthy people on day $n$, $P_{n}^{i}$ is the number of undetected contagious carriers infected for $i$ days $\left( {1 \le i \le N_{T} } \right)$. Similarly, $Q_{n}^{i}$ is the number of detected contagious carriers infected for $i$ days $\left( {1 \le i \le N_{T} } \right)$ on day $n,$ and $I_{n}$ is the number of people who were immunized. We assume that the risk of reinfection by SARS-CoV-2 after a first infection is negligible.

$N_{T}$ is the number of days a person is contagious and α is the percentage of the population tested on each day. $R_{0}$ is the number of healthy people who a contagious person contacts and infects We assume that $R_{0}$ varies over time and peaks when the virus load is maximal: 7 days after the start of infection^31,32^. In the absence of a consensus, we assume that the number of days a person is contagious is equal to the time of infection i.e. 20 days^32,33^. For all $1 \le i \le N_{T}$, $R_{0}^{i}$ is:

$$R_{0}^{i} = ~R_{0} \cdot e^{{ - \frac{1}{2}\left( {\frac{{i - 7}}{{\sqrt {20} }}} \right)^{2} }} .$$

$N$ is the total population at the start of the epidemic phase, $c$ is the multiplier for the pace of the epidemic throughout lockdown $\left( {0 \le c \le 1} \right)$, and $q$ is the same multiplier during the quarantine period $\left( {0 \le q \le 1} \right)$. $c$ and $q$ are set at 1 when there is no lockdown or quarantine. The lower the values of $~c$ or $q$, the greater the constraint which applies to the spread of the virus.

$N~$ is given by:

$$N = ~S_{n} + ~P_{n} + ~Q_{n} + ~I_{n} .$$

On transition from day $n$ to day $n + 1$, we have:

2 $$\forall ~1 \le i \le N_{T} - 1,~P_{{n + 1}}^{{i + 1}} = ~P_{n}^{i} ~\left( {1 - \alpha } \right),$$

3 $$P_{{n + 1}}^{1} = \frac{{S_{n} }}{N} \cdot \left[ {\mathop \sum \limits_{i} P_{n}^{i} \cdot R_{0}^{i} \cdot c + \mathop \sum \limits_{i} Q_{n}^{i} \cdot R_{0}^{i} \cdot q~} \right],$$

4 $$I_{{n + 1}} = ~I_{n} + ~P_{n}^{{N_{T} }} + ~Q_{n}^{{N_{T} }} .$$

$Q_{{n + 1}}^{1} = 0~$ (no quarantine on day one, test results needed).

5 $$\forall ~1 \le i \le N_{T} - 1,~\;Q_{{n + 1}}^{{i + 1}} = ~Q_{n}^{i} + P_{n}^{i} ~ \cdot \alpha .$$

We set $R_{0} = 2.2$^24^ at its peak.
